# Genomic surveillance reveals multiple introductions of SARS-CoV-2 into Northern California

**DOI:** 10.1126/science.abb9263

**Published:** 2020-06-08

**Authors:** Xianding Deng, Wei Gu, Scot Federman, Louis du Plessis, Oliver G. Pybus, Nuno Faria, Candace Wang, Guixia Yu, Brian Bushnell, Chao-Yang Pan, Hugo Guevara, Alicia Sotomayor-Gonzalez, Kelsey Zorn, Allan Gopez, Venice Servellita, Elaine Hsu, Steve Miller, Trevor Bedford, Alexander L. Greninger, Pavitra Roychoudhury, Lea M. Starita, Michael Famulare, Helen Y. Chu, Jay Shendure, Keith R. Jerome, Catie Anderson, Karthik Gangavarapu, Mark Zeller, Emily Spencer, Kristian G. Andersen, Duncan MacCannell, Clinton R. Paden, Yan Li, Jing Zhang, Suxiang Tong, Gregory Armstrong, Scott Morrow, Matthew Willis, Bela T. Matyas, Sundari Mase, Olivia Kasirye, Maggie Park, Godfred Masinde, Curtis Chan, Alexander T. Yu, Shua J. Chai, Elsa Villarino, Brandon Bonin, Debra A. Wadford, Charles Y. Chiu

**Affiliations:** 1 Department of Laboratory Medicine, University of California, San Francisco, CA, USA.; 2 UCSF-Abbott Viral Diagnostics and Discovery Center, San Francisco, CA, USA.; 3 Department of Zoology, University of Oxford, Oxford, UK.; 4 Lawrence Berkeley National Laboratory, Berkeley, CA, USA.; 5 California Department of Public Health, Richmond, CA, USA.; 6 Department of Biochemistry and Biophysics, University of California, San Francisco, CA, USA.; 7 Fred Hutchinson Cancer Research Center, Seattle, WA, USA.; 8 Brotman Baty Institute for Precision Medicine, Seattle, WA, USA.; 9 Department of Laboratory Medicine, University of Washington, Seattle, WA, USA.; 10 Department of Genome Sciences, University of Washington, Seattle, WA, USA.; 11 Institute for Disease Modeling, Bellevue, WA, USA.; 12 Department of Medicine, University of Washington, Seattle, WA, USA.; 13 Howard Hughes Medical Institute, University of Washington, Seattle, WA, USA.; 14 Department of Immunology and Microbiology, The Scripps Research Institute, La Jolla, CA, USA.; 15 U.S. Centers for Disease Control and Prevention, Atlanta, GA, USA.; 16 San Mateo County Department of Public Health, San Mateo, CA, USA.; 17 Marin County Division of Public Health, San Rafael, CA, USA.; 18 Solano County Department of Public Health, Fairfield, CA, USA.; 19 Sonoma County Department of Public Health, Santa Rosa, CA, USA.; 20 Sacramento County Division of Public Health, Sacramento, CA, USA.; 21 San Joaquin County Department of Public Health, Stockton, CA, USA.; 22 San Francisco County Department of Public Health, San Francisco, CA, USA.; 23 County of Santa Clara, Public Health Department, Santa Clara, CA, USA.; 24 Department of Medicine, Division of Infectious Diseases, University of California, San Francisco, CA, USA.

## Abstract

The COVID-19 pandemic caused by the novel coronavirus SARS-CoV-2 has spread globally, with >52,000 cases in California as of May 4, 2020. Here we investigate the genomic epidemiology of SARS-CoV-2 in Northern California from late January to mid-March 2020, using samples from 36 patients spanning 9 counties and the Grand Princess cruise ship. Phylogenetic analyses revealed the cryptic introduction of at least 7 different SARS-CoV-2 lineages into California, including epidemic WA1 strains associated with Washington State, with lack of a predominant lineage and limited transmission between communities. Lineages associated with outbreak clusters in 2 counties were defined by a single base substitution in the viral genome. These findings support contact tracing, social distancing, and travel restrictions to contain SARS-CoV-2 spread in California and other states.

The novel severe acute respiratory syndrome coronavirus 2 (SARS-CoV-2), which causes coronavirus disease 2019 (COVID-19), is a pandemic that has infected more than 3.2 million people around the world and caused more than 250,000 deaths as of May 4, 2020 (*1*), including >1.2 million cases in the United States (US) and >52,000 in California. An exponential growth in the number of cases has overburdened clinical care facilities and threatens to overwhelm the medical workforce. The reported case numbers also underestimate the true number of infections due to the limited volume of diagnostic testing to date and the presence of asymptomatic or mild cases (*2*–*4*). As a result, California, along with many other states and countries, has issued a “shelter-in-place” policy for all residents, effective March 20, 2020, and ongoing at the time of this report. These unprecedented measures have disrupted daily life significantly for ~40 million inhabitants for an indefinite period, with the potential for incurring profound economic losses (*5*).

Until late Feb 2020, the majority of infections identified in the US were related to travelers returning from high-risk countries, repatriated citizens under quarantine, or close contacts of infected patients. Community spread, in which the source of the infection is unknown, has since been documented in multiple states. In particular, Washington State has reported a series of COVID-19 cases from Jan 21 to Mar 18, following the identification of the earliest case reported in the US, WA1, on Jan 19, suggesting the presence of a persistent transmission chain in the community (*6*, *7*).

Genomic epidemiology of emerging viruses has proven to be a useful tool for outbreak investigation and for tracking virus evolution and spread (*7*–*9*). During the Ebola virus disease epidemic of 2013-2016 in West Africa, genomic analyses established that the outbreak had a single zoonotic origin (*9*), that two major viral lineages were circulating (*10*), and that sexual transmission played a role in maintaining some transmission chains (*11*). Viral genome sequencing also uncovered the route that Zika virus traveled from northern Brazil to other regions (*12*), including Central America and Mexico (*13*) and the Caribbean and US (*14*). However, real-time genomic epidemiology data of COVID-19 to inform public health interventions in California have been lacking to date.

We recently developed a method called MSSPE (Metagenomic Sequencing with Spiked Primer Enrichment) to rapidly enrich and assemble viral genomes directly from clinical samples (*15*). Here we used this method and/or tiling multiplex PCR to recover viral genomes from COVID-19 patients in Northern California and perform phylogenetic analyses to better understand the genetic diversity of SARS-CoV-2 in the US and the nature of transmission of virus lineages in the community.

We screened a total of 62 respiratory swab samples from 54 COVID-19 patients available from hospitals and clinics at University of California, San Francisco (UCSF), the California Department of Public Health (CDPH), and 8 county public health departments in Northern California (table S1). Presumptive positive cases were confirmed to be SARS-CoV-2 infected by testing using a CDC assay approved by a Food and Drug Administration (FDA) Emergency Use Authorization (EUA) on February 4, 2020 (*16*). SARS-CoV-2 genomes (>65% coverage) were recovered from 36 patients (Fig. 1A and table S2). The 36 infected patients for whom viral genomes were obtained were collected from January 29 to March 20, 2020 and spanned 9 counties in Northern California (Fig. 1B and table S2). The patient samples included (i) 11 samples collected from the Grand Princess cruise ship, during its two voyages from San Francisco to Mexico and Hawaii in February and March 2020, (ii) 3 samples from a Solano County cluster that included the first reported case of community transmission in the US with subsequent spread to two health care workers, (iii) 7 samples from Santa Clara County from a local outbreak cluster associated with workspace transmission, (iv) 3 samples from patients who contracted the infection from a sick contacts,(v) 5 samples related to domestic or international travel, and (vi) 7 samples from additional cases of community transmission.

**Fig. 1:**
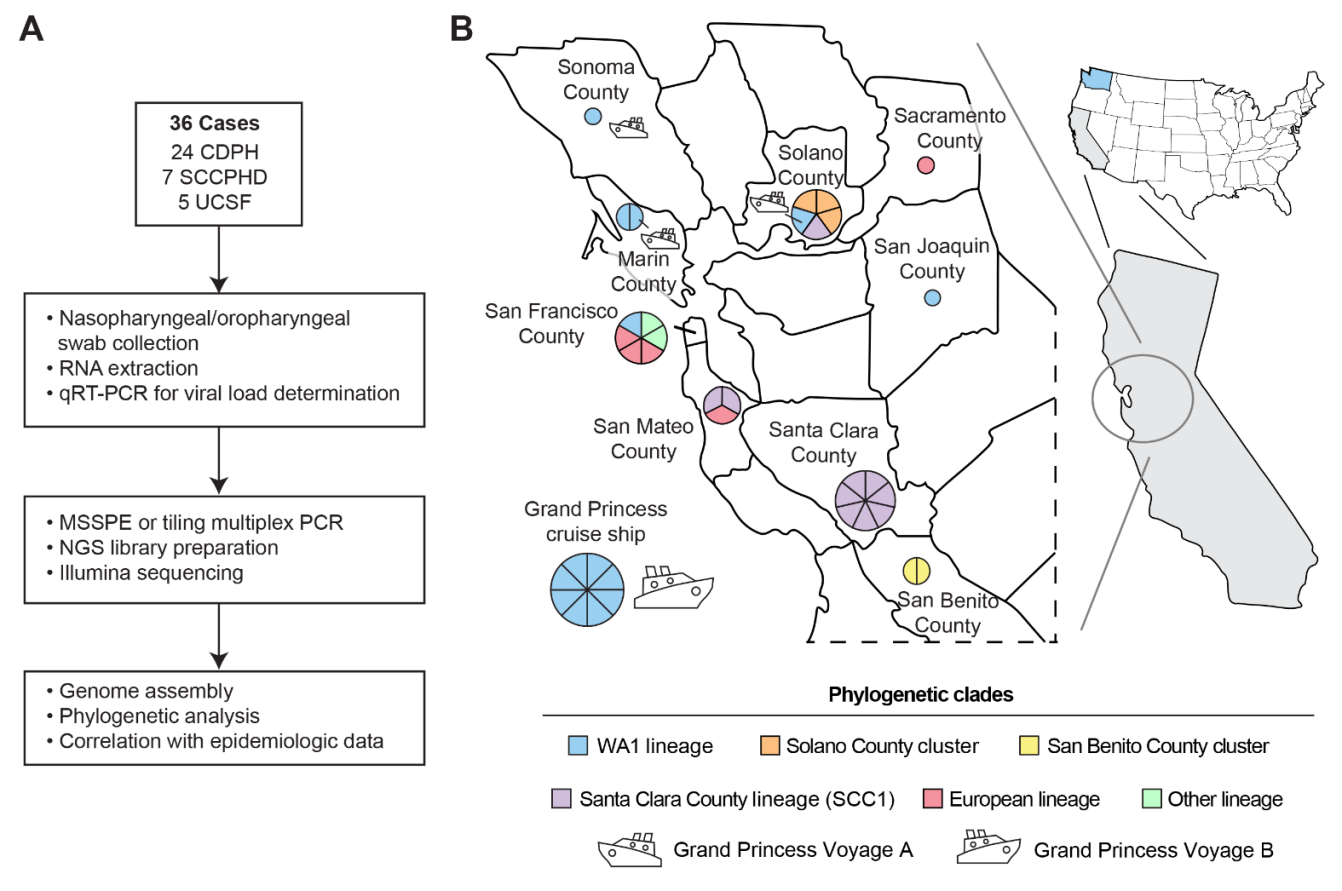
Genomic survey of SARS-CoV-2 genomes in Northern California. (**A**) Analysis workflow. (**B**) Map of the Northern California survey region divided by county. The pie charts for each county are subdivided according to the number of patients whose viral genome was sequenced, and the color corresponds to the viral lineage as determined by phylogenetic analysis. Passengers (n = 3) who were on the Grand Princess cruise ship during voyage A to Mexico and disembarked to return to their home communities are denoted by a ship icon facing left, while passengers (n = 8) aboard the Grand Princess cruise ship during voyage B to Hawaii are denoted by a ship icon facing right. Abbreviations: SCCPHD, Santa Clara County Public Health Department; CDPH, California Department of Public Health; UCSF, University of California San Francisco; MSSPE, Metagenomic Sequencing with Spiked Primer Enrichment; NGS, next-generation sequencing.

We performed MSSPE (*15*) and/or tiled multiplex PCR (*17*) on each sample to enrich for the SARS-CoV-2 RNA genome, followed by metagenomic next-generation sequencing (mNGS) of pooled and indexed samples on Illumina NextSeq, HiSeq or MiSeq instruments (*18*, *19*). The PCR cycle thresholds ranged from 15.3 to 33.4, corresponding to virus loads of 1.1 × 10^4^ − 2.7 × 10^8^ copies/mL (fig. S1 and table S2). An average of 31 million (interquartile ratio, IQR, 23-57 million) and 2.2 ± 0.2 million reads were generated per sample for using MSSPE and tiling multiplex PCR respectively, and virus genomes were assembled by mapping to reference genome NC_045512 (Wuhan-Hu-1). The assembly yielded 34 SARS-CoV-2 genomes with genome coverage >65% and these were included in the study. An additional two genomes sequenced from samples of a returning traveler from Wuhan, China and a household contact collected on January 29th by the CDC (CA3 and CA4) were also included in the analysis. The median coverage achieved across all samples was 97.7% (IQR 90.4.0%-99.7%).

Phylogenetic analysis revealed that the 36 SARS-CoV-2 genomes from California generated in this study were dispersed across the evolutionary tree of SARS-CoV-2 that was built from 789 worldwide genomes deposited into GISAID as of March 20, 2020 (Fig. 2A). The 36 genomes included 14 in the Washington State (WA1) lineage, 10 in a lineage associated with the Santa Clara County outbreak cluster (henceforth referred to as the SCC1 lineage), 3 from a Solano County cluster of 3 individuals, 5 related to lineages circulating in Europe and New York, and 4 related to early lineages from Wuhan or other regions of China (including 2 patients from San Benito County with identical genomes) (Figs. 1, 2A, and 3 and table S2).

**Fig. 2:**
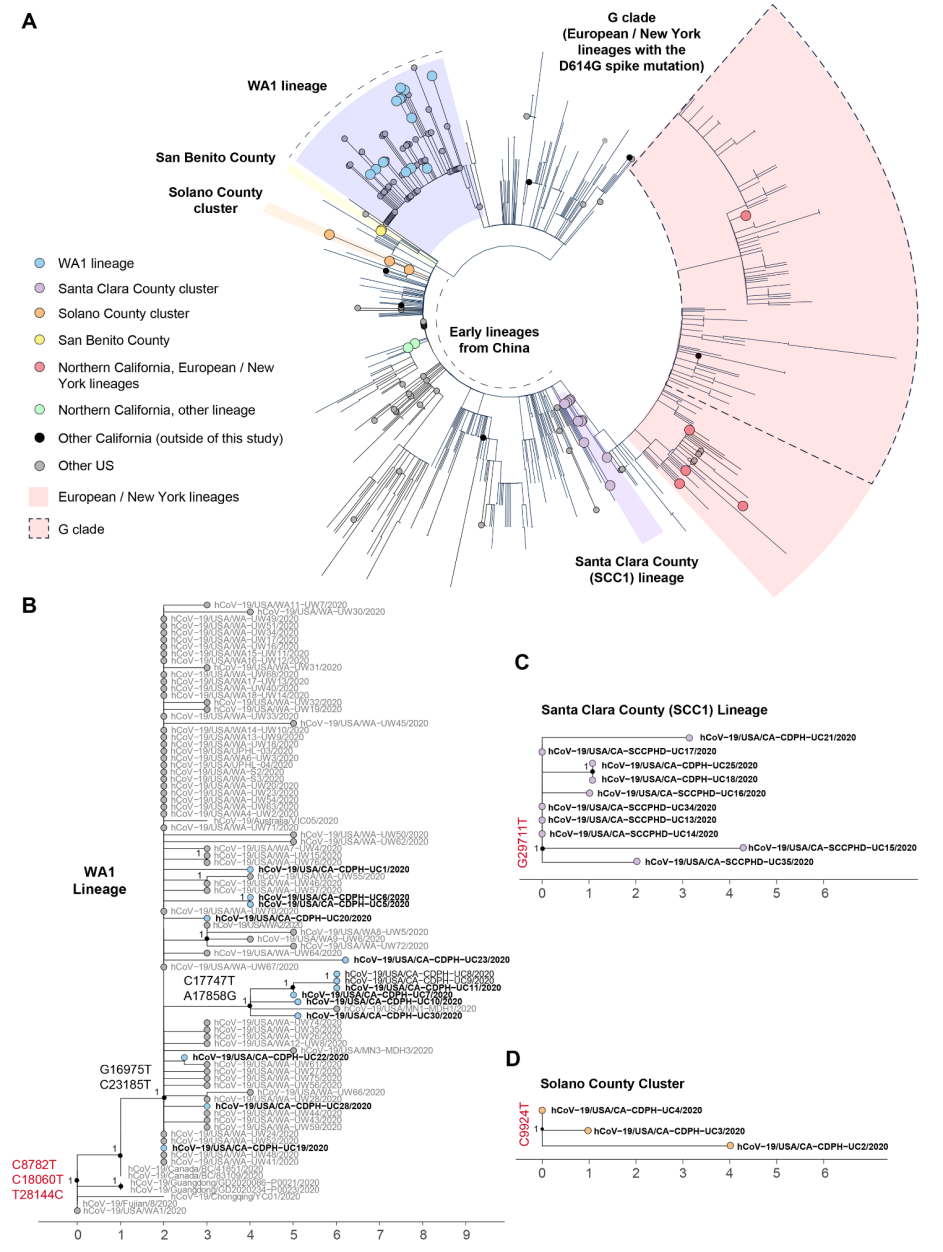
Phylogeny of SARS-CoV-2 lineages in California. (**A**) Phylogenetic tree of 753 SARS-CoV-2 genomes from GISAID (till Mar 20, 2020) along with the 36 genomes in this survey (tree file attached in supplementary material). Genomes from Northern California sequenced in the current study are denoted with colored circles, while other genomes sequenced from California and from other states in the US are denoted with black and gray circles, respectively. The name of each lineage or outbreak cluster is shown next to the arc line. (**B**) Phylogenetic subtree corresponding to the WA1 lineage. This subtree was reconstructed from 88 SARS-CoV-2 genomes after removal of ambiguous nucleotide sites that had generated low-coverage artifacts (see text). The WA1 virus from Washington state (first case in the US) is at the root of the subtree along with a virus sequenced in China. The UC19 virus (from a Grand Princess voyage A passenger) is basal to the viruses sequenced from crew members and passengers on voyage B. (**C**) Zoomed view of the SCC1 lineage associated with the Santa Clara County outbreak cluster. (**D**) Zoomed view of the Solano County cluster. The x-axis shows the number of base substitutions relative to the root of the phylogenetic tree. The key SNVs defining a lineage or cluster are shown in red text. Bootstrap values (converted from the approximate likelihood ratio test, or aLRT score) are displayed at each node, with a value of 1 indicating 100% support.

**Fig. 3:**
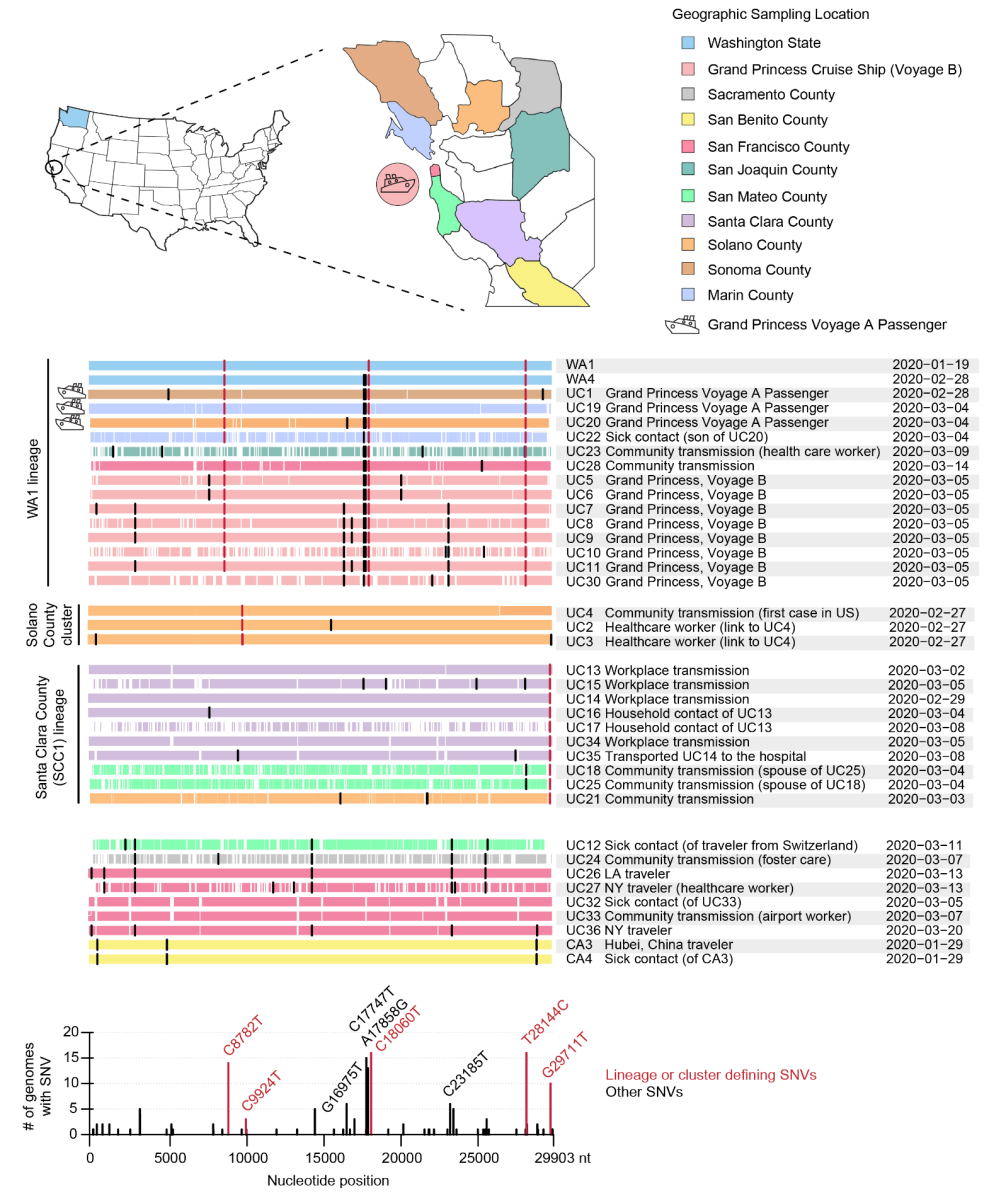
Multiple sequence alignment of all SARS-CoV-2 genomes reported across 9 Northern California counties and the Grand Princess cruise ship. Single nucleotide variants (SNVs) with respect to the reference genome (NC_045512) are shown as vertical red and black lines for lineage defining SNVs and other SNVs, respectively. Cases that are part of the WA1 lineage include the first case of COVID-19 infection (WA1) in the US, 8 passengers and crew members aboard the Grand Princess cruise ship during its second trip (voyage B), and 3 individuals surveyed from 3 Northern California counties as passengers on the ship’s first trip (voyage A). The three SNVs C8782T, C18060T, and T28144C define the WA1 lineage, and the two SNVs C17747T, A17858G are common to Grand Princess passengers and crew. Viruses from voyage B passengers and crew share SNVs G16975T and C23185T that are lacking in viruses from voyage A passengers. Single SNV variants C9924T and G29711T define the lineages from Solano County and Santa Clara County, respectively. The putative epidemiological link and sample collection date are shown beside the sequence alignment.

A large outbreak was associated with travel on the US Grand Princess cruise ship (with at least 78 confirmed positive cases out of 469 tested) as of March 26 (*20*). The Grand Princess undertook two consecutive voyages from San Francisco (voyage A to Mexico on February 11 − 21 and voyage B to Hawaii on February 22 − March 4), with much of the same crew and a shared subset of passengers. Samples from 11 infected patients were sequenced, 3 of whom had been on voyage A and became sick after returning to their home county, and 8 from crew members and passengers aboard the cruise ship on voyage B. Importantly, all 11 available sequenced genomes from the Grand Princess were part of the WA1 lineage (Fig. 2, A and B, and Fig. 3). In addition to sharing 3 single nucleotide variants (SNVs) that define WA1 (C8782T, C18060T, and T28144C), the sequences from cruise ship passengers and crew also shared two additional SNVs, C17747T and A17858G common to nearly all WA1 sequences sampled from Washington and California but not the basal WA1 case (Figs. 2B and 3).

The WA1 case was reported on January 19 (*6*), and thus substantially predated the voyages of the Grand Princess cruise ship (*7*, *20*). In addition, 6 of 8 passengers on voyage A (UC 7 −11, 30) carried at least 2 new mutations (G16975T and C23185T) not observed in UC1, UC19, and UC20, who were all on the first cruise (Fig. 3). This suggested that the virus from UC19 could be basally positioned relative to the cruise ship strains from voyage B, and that COVID-19 infections associated with voyage A may have been passed onto passengers and crew on voyage B. However, the initial WA1 subtree extracted from the global maximum-likelihood phylogenetic tree did not place UC19 basal to sequences from voyage B passengers due to artifacts from shared areas of low coverage (fig. S2). To establish a more accurate tree topology, we therefore reconstructed a new phylogenetic subtree of the WA1 lineage after excluding all ambiguous sites. In this new subtree (Fig. 2B), UC19 is basal to all other California genomes within the WA1 lineage. In addition, among the sequences from patients on voyage B, UC5 and UC6 group together, while UC7-11 and UC30 group together with a sequence sampled in Minnesota.

The chronology and phylogeny of the cruise ship outbreak, along with the predominance of the WA1 lineage in Washington State (*7*), suggest that the virus on the Grand Princess likely came from Washington State, although the cases may also have originated from a different region in which the WA1 strain is circulating. In addition to passengers and crew members aboard the Grand Princess, virus genomes sampled from three cases of community transmission in different counties of the Bay area (UC22, UC23 and UC28) were also of the WA1 lineage. UC22 was the son of an infected Grand Princess passenger (UC20) on voyage A and most likely contracted the virus from household contact. The UC23 and UC28 cases may also reflect transmission from disembarking Grand Princess passengers on voyage A, or pre-existing circulation of the WA1 strain in the community.

Three patients examined in this study (CA3, CA4, and UC12) had COVID-19 infections associated with international travel or exposure to international travelers. CA3 corresponds to a resident of San Benito County who became sick shortly after returning from Wuhan, China in late January. The sequence of his SARS-CoV-2 genome is identical to that of CA4, a household contact who was also infected with the virus. Their viral genomes were found to be closely related to early lineages from China (Fig. 2A and data S1). UC12 had a prolonged exposure to a known positive traveler from Switzerland while attending a conference. The genome from UC12 fell within a lineage containing many sequences from European residents or travelers from Europe (Fig. 2A). Interestingly, four additional genomes (UC24, UC26, UC27 and UC36) were also grouped within the European lineage. UC27 and UC36 were both diagnosed shortly after returning to California from New York, consistent with reports that the New York outbreak that began in March 2020 originated with travelers coming from Europe (*21*, *22*). UC26 also reported domestic travel from Los Angeles, while UC24 had no known travel history.

In Santa Clara County, we sequenced 7 genomes from individuals who were part of a local outbreak of COVID-19 at a large workplace facility with multiple employers, large areas of shared space, and heavy pedestrian traffic. The genomes all shared the G29711T SNV that defines the SCC1 lineage (Figs. 2C and 3). Four employees (UC13, UC14, UC15, and UC34) had dates of symptom onset within two weeks of each other, although they did not know each other. UC16 and UC17 were family members of UC13 and lived in the same residence, while UC35 transported UC14 to the hospital via emergency medical services. Notably, the genomes from a Solano county resident (UC21) and a San Mateo couple (UC18 and UC25) were also placed in the SCC1 lineage, suggesting possible spread to different counties. Further epidemiological investigation found that UC21 had visited a merchant in Santa Clara, during which he likely became infected.

In Solano County, a small cluster of 3 cases included the first reported instance of community transmission in the US on February 26 (UC4) (Figs. 2D and 3). The two other cases (UC2 and UC3) were healthcare workers who were taking care of patient UC4 and likely contracted the disease in the hospital, consistent with transmission of the disease from patient to health care providers (*23*). The genomic epidemiology of the COVID-19 cases associated with community spread studied here do not show any predominant SARS-CoV-2 lineage circulating in Northern California. In California, multiple recent and unrelated introductions of SARS-CoV-2 into the state via different routes appear to give rise to the diversity of virus lineages reported in this study, with no single predominant lineage observed. We note that this does not exclude the possibility of cryptic transmission of multiple lineages in California simultaneously, as the current level of sampling is not dense enough to confidently estimate the dates of the seeding events, nor the subsequent periods of cryptic transmission before a lineage was identified.

There is growing evidence that the WA1 is now an established lineage of SARS-CoV-2 in the US. Here we found that viruses in the WA1 lineage from Grand Princess cruise ship passengers as well as from residents of several Northern California counties. In addition, WA1 lineage viruses have been identified in COVID-19 cases from many states including Minnesota, Connecticut, Utah, Virginia, and New York (*24*, *25*). The early date and basal phylogenetic position of the WA1 virus make it likely that the direction of dissemination was from Washington State to California and other states; however, this conclusion could change if further genomic sampling in the US revealed additional virus genetic diversity. Notably, SARS-CoV-2 sequences from Connecticut (*25*) and British Columbia, Canada (Fig. 2B) are positioned close to the root of the subtree containing the WA1 sequences, raising the possibility that the virus may not have been first introduced into the US via Washington State.

SARS-CoV-2, like other coronaviruses, contains a nonstructural gene with proofreading activity (*26*). Consequently, the virus evolves more slowly than many other human RNA viruses, on the order of 1 to 2 DNA base substitutions a month across its ~29 kB genome (*27*). Thus, only 1-3 SNVs in general are needed to define a distinct lineage. The WA1 lineage consists of 3 key SNVs, C8782T, C18060T, and T28144C, while the SCC1 lineage associated with the Santa Clara County cluster and the Solano County cluster are each defined by only one SNV, G29711T and C9924T, respectively (Figs. 2 and 3).

Our epidemiological and genomic survey of SARS-CoV-2 has several limitations. First, this initial analysis represents a relatively sparse sampling of cases. Undersampling of virus genomes is due in part to the high proportion of cases (80%) with asymptomatic or mild disease (*2*–*4*) and limited diagnostic testing for COVID-19 infection to date in California and throughout the US. Second, the majority of samples analyzed were obtained from public health laboratories and thus may not be representative of the general population. Finally, phylogenetic grouping of viruses from different locations, such as Washington State and California in the same WA1 lineage, does not prove the directionality of spread. Despite this, our study shows that robust insights into COVID-19 transmission are achievable if virus genomic diversity is combined and jointly interpreted with detailed epidemiological case data. In particular, we found that a returning traveler from New York was infected with a lineage circulating widely in Europe, thus suggesting an association between the New York outbreak and intercontinental travel to and from Europe before this was widely recognized (*21*, *22*).

Public health containment measures such as isolation and contact tracing, as performed in the Solano County and Santa Clara County outbreak clusters, become more difficult to maintain once a lineage becomes established in the community. Our data suggest concerning trends in this direction, such as the association between the WA1 lineage and community-acquired COVID-19 cases in several counties of Northern California, and a virus from the SCC1 lineage detected in residents of neighboring San Mateo and Solano County. Social distancing interventions, such as the “shelter-in-place” directive that was issued by the governor of California on March 20, 2020, may assist in stemming spread from community to community. Interstate dissemination of SARS-CoV-2 lineages has also been demonstrated coast-to-coast between Washington State and Connecticut (*25*), and from domestic and international travel into the San Francisco Bay Area in the current study. Suspension of non-essential travel may thus be necessary to prevent ongoing importation of new cases in California and other states.

## Supplementary Materials

science.sciencemag.org/cgi/content/full/science.abb9263/DC1 Materials and Methods Figs. S1 and S2 Tables S1 to S5 Data S1 References (*31*–*39*)
